# Prosodic Cues Support Inferences About the Question’s Pedagogical Intent

**DOI:** 10.1162/opmi_a_00192

**Published:** 2025-02-16

**Authors:** Igor Bascandziev, Patrick Shafto, Elizabeth Bonawitz

**Affiliations:** Harvard Graduate School of Education, Cambridge, MA, USA; Rutgers University–Newark, Newark, NJ, USA

**Keywords:** pedagogical intent, questions, prosody

## Abstract

Questions may be asked with an intent to acquire new information from the recipient (i.e., information-seeking questions) or with the intent to teach (i.e., pedagogical questions). Understanding how the questions’ recipients infer the intent of questions is important, because the recipients’ inferences have important consequences for reasoning and learning. In the present series of studies, we tested the hypothesis that i) askers use prosodic cues—an ever-present signal—to encode information-seeking and pedagogical intent both in deliberate and spontaneous speech and that ii) adults and children can draw appropriate inferences about the question’s intent on the basis of prosody alone. In Experiments 1 and 2, we found that naïve adult listeners and children aged 5 years and above have the capacity to explicitly identify which asker has an intention to teach on the basis of prosody alone. In Experiment 3, we found that parents’ spontaneous speech in pedagogical or information-seeking contexts is appropriately recognized by naïve listeners as pedagogical or information-seeking. Thus, the intent of pedagogical and information-seeking questions is acoustically encoded by askers, and it can be appropriately decoded by recipients.

## INTRODUCTION

At least since the time of Plato, it has been understood that asking questions can be an important tool in the service of discovery, learning, and teaching (Plato, 1892). Receiving questions can help one to recognize one’s ignorance, which can in turn motivate and shape the process of learning (Csibra & Gergely, 2009; Yu et al., 2018). We call these pedagogical questions. However, questions also serve a rhetorical or an indirect request function, and most frequently, questions serve to gain information from the recipient (Aguirre et al., 2022; Ronfard et al., 2020). We call these information-seeking questions. That there are at least two importantly different categories of questions (i.e., pedagogical and information-seeking) raises the issue of how the two are differentiated by askers and recipients.

One way of differentiating between pedagogical and information-seeking questions is by relying on cues such as the content and the context in which the question was asked (Clark, 1977; Frank & Goodman, 2012; Grice, 1978, 2002; Sperber & Wilson, 1995). If it is known that the asker knows the answer to the question, then the likely reason for asking it is to initiate learning in the recipient. An important difference between a pedagogical and non-pedagogical context is that in a pedagogical context, the learner infers that the teacher is *choosing* data (or questions) as a knowledgeable and helpful person (Bonawitz & Shafto, 2016). That is, the data or the question are designed to help the recipient’s learning. The learner’s inference that the question is asked with a pedagogical intent would then prompt a different way of thinking about the question compared to receiving an information-seeking question. Indeed, when the pedagogical context is explicit, the recipient shows differential behavior (Bonawitz et al., 2020) and superior learning outcomes compared to when they receive an information-seeking question (Jean et al., 2019; Yu et al., 2018). For example, when 4- to 6-year-olds are asked by a knowledgeable person (i.e., a person who made the toy) “What does this button do?” children activated the target function and produced more unique actions on the toy compared to children who heard the same question asked by a naïve asker (Yu et al., 2018). Thus, the recipient’s inference about the intent of the question, drawn from explicit information about the asker’s knowledge state, has important consequences for learning. However, while useful in laboratory experiments to show the power of pedagogical questions, explicit knowledge of other’s knowledge is likely rare outside the lab.

Although explicit signals about the questioner’s knowledge state are rare, questions in day-to-day experiences are ubiquitous, and seem to play an important role in children’s development. In Western societies, even young infants begin receiving questions as early as 5-months of age (Bornstein et al., 1992). Furthermore, almost half of the utterances that 12- to 27-month-olds hear are questions (Newport et al., 1977). At school, children hear 300 to 400 questions per day (Levin & Long, 1981). Presumably, many, but not all of those questions are pedagogical. Indeed, one study that investigated questions recorded in the CHILDES database found that pedagogical questions are common, but they vary across SES and different historical eras (Yu et al., 2019). That study coded pedagogical and information-seeking questions on the basis of indirect evidence about the parent’s knowledge state. However, aside from the recipients’ assumptions about the knowledge state of the askers and the explicit signals provided by the askers about their knowledge state, it is not clear how parents and teachers *mark* the intent of pedagogical questions, and how children draw inferences about the question’s intent.

Although factors such as the content of question, the status of the asker, or the context in which the question is asked could all be powerful cues, they are most likely ambiguous and insufficient for inferring the question’s intent under many circumstances. That is, a question with the same content, asked by the same speaker in the same context could be a pedagogical or information-seeking question. For example, a parent, who is typically knowledgeable, in the context of playing with her child may ask: “Why did you put this here?” as an information-seeking question (i.e., to learn about the intent of the child), or as a pedagogical question (i.e., to signal a pedagogical episode that there is something important to be learned from the child’s action of putting the object in a particular place). The parent is unlikely to offer additional clarifying content to the question such as “I really don’t know the answer” or “I want you to learn from this.” Thus, even if a questioner is typically in a pedagogical role and known to be a knowledgeable helpful other, they cannot be assumed to always intend to ask pedagogical questions. In sum, most cues about the intent of the question are likely to be ambiguous and uninformative in everyday communication.

Understanding the intent of a question has real consequences for communicative inference and learning. It is thus important to understand what cues learners might use to disambiguate them. In the present study, we investigate a potential mechanism that is always available in natural speech for communicating pedagogical intent of questions, namely, the prosody of speech. Prosody is a plausible hypothesized mechanism for several reasons. First, unlike many other cues, prosody is always present in speech in neurotypical individuals with no pathological changes in the laryngeal muscles (Chung et al., 2018). Second, although there is a great information redundancy between context, the content of speech, and prosody, prosody seems to carry information over and above the context and content of speech (Wolf et al., 2023). Third, prosody can carry information about *intent specifically* (Hellbernd & Sammler, 2016). For example, the dynamic and summary prosodic features of infant-directed speech seem to carry information about various pragmatic categories, such as drawing the infant’s attention, providing comfort, or approval (Katz et al., 1996; Kitamura & Burnham, 2003; Papoušek et al., 1991; Schachner & Hannon, 2011). Interestingly, the communicative intent is more recognizable if delivered in infant-directed speech than in adult-directed speech (Fernald, 1989). Finally, although prosody may seem like a subtle cue for young children, as noted above, even very young infants are sensitive to different registers of speech such as child- and adult-directed speech (Saint-Georges et al., 2013). Taken together, we hypothesize that i) the speakers’ pedagogical and information-seeking intent of questions is marked by differentiating prosodic cues both in deliberate and spontaneous speech, and ii) adults and children can draw appropriate inferences about the question’s pedagogical or information-seeking intent on the basis of prosody alone.

### Prosody: A Plausible Mechanism for Communicating Pedagogical Intent

Unlike content, status, and assumed knowledge-state, the prosody of speech is a reliably present signal that can disambiguate intent. Although there is a large literature on the prosody of questions (e.g., rhetorical, information-seeking, indirect requests, etc.; Banuazizi & Creswell, 1999; Bartels, 1999; Braun et al., 2019; Han, 2002; Trott et al., 2023), there are no studies that have documented the prosody of pedagogical questions. One particular kind of prosody that has been investigated extensively in the context of how the prosody of speech can facilitate learning, is the prosody of child-directed speech (Eaves et al., 2016; Fernald, 1985; Fernald & Simon, 1984; Nencheva et al., 2021; Rowe, 2008; Thiessen et al., 2005). Indeed, several studies have shown that the prosody of child-directed speech is also interpreted as an ostensive signal by learners (Gergely et al., 2007; Senju & Csibra, 2008). However, child-directed speech cannot be a reliable signal of pedagogy. This is so because adults use child-directed speech across various situations that do not entail teaching about the world. Also, when children are older, adults no longer use child-directed speech even when they teach (Liu et al., 2009). Therefore, whether specific prosodic cues differentiate pedagogical from information-seeking questions, beyond and above the distinction of child-directed and adult-directed speech, remains unclear.

Another reason why the prosody of language is a plausible mechanism for differentiating between pedagogical and information-seeking questions is its capacity to carry information about the speaker’s epistemic state. For example, adults use prosodic markers to signal their confidence when answering questions (Smith & Clark, 1993). Developmentally, the ability to detect disbelief explicitly in the prosody and facial gestures seems to emerge between the ages 3 and 5, where 5-year-olds do better than 3-year-olds, having both visual and audio cues helps more than having a single cue (i.e., visual or audio), and children are comparably effective at using each cue on its own when inferring disbelief in the speaker (Armstrong et al., 2018). The production of prosodic cues of uncertainty also seems to emerge between the ages 3 and 5, although this ability continues to develop in later childhood (Hübscher et al., 2019).

The prosody of questions may also communicate the knowledge state of the asker. One particular prosodic cue that seems to communicate the askers’ knowledge state is the falling or rising intonation at the end of the utterance. A canonical falling intonation of declarative statements in English is consistent with speakers’ commitment to the truth of the proposition. A rising, non-canonical intonation, however, seems to signal uncertainty about the truth of the statement (Gunlogson, 2004). Similarly, a canonical rising intonation of Yes–No questions signals an information-seeking intent, whereas a non-canonical falling intonation signals a different intent (Hedberg & Sosa, 2011). The intonation at the end of Wh- questions is reversed. A falling intonation in a Wh- question is canonical and it signals that the asker seeks information. Conversely, a rising intonation is non-canonical, and it signals that the asker has partial knowledge of the answer (Hedberg et al., 2010). Consider the following dialog reported in Hedberg and Sosa (37, p. 7): “A: Did you hear? We have a new department secretary. B: Yes, I heard. What’s her name? (noncanonical rising intonation). A: Gina. B: No, that’s not it. It starts with an S.” The rising intonation at the end of the question “What’s her name?” is consistent with B’s disagreement that the new person’s name is Gina. If, however, speaker B asked the question “What’s her name” with a canonical falling intonation, then disagreeing with the answer would be unusual. This indicates that a non-canonical intonation at the end of an utterance signals a noncanonical knowledge state of the speaker for that type of an utterance. It indicates lack of knowledge in the case of declarative statements [36] and some knowledge in the case of interrogative utterances (Hedberg & Sosa, 2011). Considering that pedagogical questions are asked by knowledgeable askers, it stands to reason that they too would have noncanonical intonation.

Taken together, this prior research suggests that prosody can serve as a critical disambiguating signal in pedagogical and information-seeking questions. However, whether the adult speakers’ pedagogical and information-seeking intent of questions is marked by differentiating prosodic cues both in deliberate and spontaneous speech, and ii) whether adults and children can draw appropriate inferences about the question’s pedagogical or information-seeking intent on the basis of prosody alone is still unclear. Across three experiments, we investigated the production and perception of pedagogical and information-seeking prosodic cues in questions. In Experiments 1 and 2, we investigated whether naïve listeners, including children, can explicitly infer the pedagogical or information-seeking intent of a question on the basis of prosody alone, where the prosody of the questions was deliberately manipulated. In Experiment 3, we investigated whether parents, during parent-child interactions, spontaneously produce prosodic cues that differentiate pedagogical from information-seeking questions, and whether those spontaneously produced prosodic cues can be accurately differentiated by naïve adult listeners.

## EXPERIMENTS, RESULTS, AND DISCUSSION

### Experiment 1a: Inferring the Questions’ Intent

In Experiment 1a, we asked whether adult naïve listeners could reliably differentiate between pedagogical and information-seeking questions. The stimuli for this experiment were developed by asking 8 (four women and four men) native speakers of American English, coming from diverse ethnic and cultural backgrounds, and with extensive experience interacting with children (i.e., as parents or developmentalists), to record 100 short questions (e.g., How do dolphins sleep?). All audio stimuli used in Experiment 1a could be found at https://osf.io/d74vt/. Each question was asked four ways: with pedagogical and information-seeking intent, and both in an adult-directed and a child-directed voice (this produced a total of 400 question utterances per speaker). The age of the imagined child was 4 or 5 years old. This design allowed for a direct comparison of the same questions, recorded as pedagogical or information-seeking with an adult- or child-directed voice. The recorded questions were administered to a sample of 128 English speakers in the US on Mechanical Turk. Each participant heard 100 questions (25 adult-directed pedagogical, 25 adult-directed information-seeking, 25 child-directed pedagogical and 25 child-directed information-seeking, spoken by a single speaker and presented in a randomized order). Participants were asked to rate the audio of each question as: a) A pedagogical question or b) An information-seeking question, as well as: a) a child-directed question or b) an adult-directed question. Each answer that participants provided on each of the two questions was scored as 1 = accurate or 0 = inaccurate. This allowed us to compute the mean accuracy score (i.e., the mean proportion of times participants answered correctly) over all utterances for each of the two questions (i.e., pedagogical vs. information-seeking and child-directed vs. adult-directed).

The primary goal was to investigate whether listeners’ ratings of pedagogical or information-seeking prosody align with the speakers’ intentions of generating pedagogical or information seeking questions. The raw data are available at https://osf.io/d74vt/. The listeners were able to distinguish intention from prosody alone. The average accuracy score for discriminating pedagogical from information-seeking questions (i.e., the mean proportion of questions classified correctly), collapsed across child and adult-directed speech was *M*_*PQ*/*ISQ*_ = .58, which is significantly above chance level performance (*t*(127) = 6.97, *p* < .001, *d* = .62).

Replicating prior findings, we additionally found that participants could also discriminate between child-directed and adult-directed speech, collapsing across pedagogical and information-seeking questions. The average accuracy score for discriminating child-directed from adult-directed speech was *M*_*CD*/*AD*_ = .56, which is significantly different from chance performance (*t*(127) = 7.42, *p* < .001, *d* = .66).

**Figure 1.**
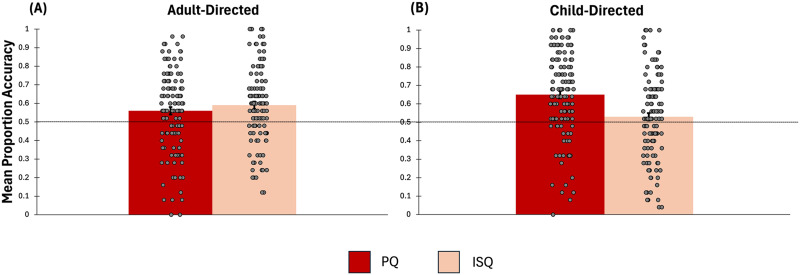
The mean proportion accuracy for discriminating pedagogical and information-seeking questions is above chance performance. The datapoints represent the participants’ accuracy scores (i.e., the proportion of accurate judgments made by each participant). Panel A represents discrimination of pedagogical from information-seeking questions within adult-directed speech. Panel B represents discrimination of pedagogical from information-seeking questions within child-directed speech. Error bars are standard errors.

Figure 1 reveals some asymmetry in the accuracy of detecting pedagogical or information-seeking questions within different categories of speech (child vs. adult directed). For example, whereas the accuracy score for pedagogical questions within child-directed speech was fairly high (*M* = .65), the accuracy score for information-seeking questions within child-directed speech was not different from chance (*M* = .53). There are at least two possible interpretations of this result. One is that some (but not all) prosodic features of pedagogical questions overlap with the prosody of child-directed speech, while some (but not all) features of information seeking questions overlap with the prosody of adult-directed speech. Another, not mutually exclusive possibility, is that listeners might have an expectation that pedagogical questions are typically directed to children, and information seeking questions are typically directed to adults. A signal Detection analysis of the Hits and False Alarms (where the pedagogical prosody is the signal and the information-seeking prosody is the absence of signal) found that within adult-directed speech the sensitivity index *d*′ was .38 and within child-directed speech, the sensitivity index *d*′ was .46. These indexes are similar to each other (i.e., a .08 of a standard deviation difference) and both are smaller than a ½ standard deviation, suggesting that there is a big overlap between the signal present and signal absent distributions. Moreover, this analysis showed that listeners are applying a conservative criterion (with lower hits and false alarms) when listening to adult-directed speech and they apply a liberal criterion (with higher hits and higher false alarms) when listening to child-directed speech. This finding suggests that listeners need less evidence to decide that a question is pedagogical when it is within child-directed speech than when it is within adult-directed speech, which is consistent with the claim that listeners are biased to think that child-directed speech is pedagogical.

Taken together, these findings show that the pedagogical and information-seeking intent of a question can be encoded in the prosody of the question, and naïve adult listeners can successfully recognize the pedagogical or information-seeking intent of the speaker in the prosody of speech. Importantly, this is a stringent test of our hypothesis considering that all of the context has been stripped from the stimuli and the only information available to participants was the auditory information contained in the speech.

One point that is not clear from these findings, is whether participants relied on prosody alone (i.e., the patterns of stress and intonation) or they relied on a combination of prosody and syntactic and semantic cues. One possibility is that prosodic cues—e.g., contrastive stress or particular pitch on particular words (e.g., interrogative pronouns or auxiliary verbs)—could differentiate pedagogical from information-seeking questions. The alternative is that the prosodic cues are an autonomous signaling system that is independent of the syntax and the semantics of the question (Bolinger, 1958; Selting, 1992). To investigate this, we asked if English speaking adults could differentiate pedagogical from information-seeking questions in a language they do not understand, thus removing access to syntactic and semantic cues.

### Experiment 1b: Inferring the Questions’ Intent in a Foreign Language

In Experiment 1b, we asked whether adult naïve speakers of native American English could reliably differentiate between pedagogical and information-seeking questions when the questions are recorded in Macedonian language. We chose Macedonian because it is unlikely to be known by the majority of participants from our US sampled pool (significantly less than 1%), and it applies different syntactic, stress, and rhythm patterns in speech than American English, but has similar intonation profiles. The stimuli for this study were the exact same questions of study 1a but translated into Macedonian language. All questions were recorded in Macedonian language and prosody by two (1 man and 1 woman) native speakers of Macedonian language who work and live in the US. The stimuli are available at https://osf.io/d74vt/. As in Experiment 1a, the recorded questions were administered to a sample of 128 English speakers in the US on Mechanical Turk. Each participant received 100 questions and was asked to rate them as: a) A pedagogical question or b) an information-seeking question, as well as: a) A child-directed question or b) An adult-directed question. Each answer that participants provided on each of the two questions was scored as 1 = accurate or 0 = inaccurate. This allowed us to compute the mean accuracy score (i.e., the mean proportion of times participants answered correctly) over all utterances for each of the two questions (i.e., pedagogical vs. information-seeking and child-directed vs. adult-directed).

**Figure 2.**
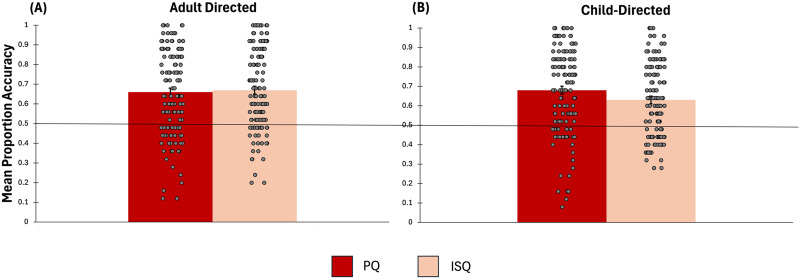
The mean proportion accuracy for discriminating pedagogical and information-seeking questions is above chance performance. The datapoints represent the participants’ accuracy scores (i.e., the proportion of accurate judgments made by each participant). Panel A represents discrimination of pedagogical from information-seeking questions within adult-directed speech. Panel B represents discrimination of pedagogical from information-seeking questions within child-directed speech. Error bars are standard errors.

Participants were able to distinguish pedagogical from information seeking intent in non-English questions. The average accuracy score for discriminating pedagogical from information-seeking questions was *M*_*PQ*/*ISQ*_ = .66, and it is significantly different from chance performance, *t*(127) = 11.7, *p* < .001, *d* = 1.03). Similarly, the average accuracy score for discriminating child-directed from adult-directed speech was *M*_*CD*/*AD*_ = .65 and it is also significantly different from chance performance (*t*(127) = 13.92, *p* < .001, *d* = 1.25). Figure 2 presents the accuracy scores within child- and adult-directed speech.

These results replicate the results of Experiment 1a. Further, they confirm that naïve adult listeners can discriminate between pedagogical and information-seeking questions both within child-directed and within adult-directed speech on the basis of prosody alone, in the absence of any syntactic or semantic cues. It is important to note, however, that the mean accuracy of Experiment 1b should not be compared to the mean accuracy of Experiment 1a, not least because the stimuli were generated by different individuals. We note that how distinguishable pedagogical from information-seeking questions are (or for that matter how distinguishable child-directed from adult-directed speech is) varies from one speaker to another. Having stimuli generated by fully bilingual speakers in two languages may serve as a better basis for direct comparisons in future work.

The findings of Experiments 1a and 1b have at least two limitations. First, each participant was asked to discriminate between pedagogical and information-seeking questions produced by a single speaker. Therefore, it is not clear if such discrimination would be possible on a one-off basis with multiple speakers who naturally differ across many prosodic dimensions. Second, the participants in Experiments 1a and 1b were adults. Therefore, it is not clear if young children can explicitly differentiate between pedagogical and information-seeking prosody. If prosody is a viable signal that can alert children to the pedagogical nature of some questions, then we should expect to find that children are able to differentiate questions on the basis of prosody alone. Furthermore, since the intent of speakers varies naturally over the course of a conversational episode, we should expect that children would be able to detect different intent within the same speaker on a one-off basis. We tested these hypotheses in Experiment 2.

### Experiment 2: Children Can Explicitly Identify Pedagogical Intent on the Basis of Prosody Alone

In Experiment 2, we asked whether 4-, 5- and 6-year-old children, as well as adults, can differentiate prototypical exemplars of pedagogical and information-seeking questions on the basis of prosody alone. We chose those ages because prior evidence has shown that 4- and 5-year-olds are able to differentiate between pedagogical and information-seeking questions when the knowledge state of the asker is marked explicitly (e.g., Yu et al., 2018). To answer the question if listeners can differentiate the intent of questions on the basis of prosody alone, participants were presented with 24 contrasts where within each contrast, one individual spoke with a pedagogical prosody and the other individual spoke with an information-seeking prosody. All questions in the 24 contrasts were spoken with a child-directed prosody. Examples of the stimuli are available at https://osf.io/d74vt/. Participants were told that they will hear the same question being asked by two different people. One person knows the answer to the question and is teaching about the question’s topic; the other person does not know the answer and is still learning about the topic. The task was to listen to each question and guess whose question was intended to be pedagogical.

The pre-registration details are available at https://aspredicted.org/yxj4-z6fv.pdf. In the initial pre-registration, we stated that we will explore age differences. However, age differences were not predicted, and it was not predicted that 4-year-olds will perform at chance level. After collecting data from 4- and 5-year-olds, which indicated age differences and chance-level performance of the 4-year-olds, we pre-registered additional data collection from 6-year-old children, which is available at https://aspredicted.org/tp6v-vpb2.pdf. The raw data are available at https://osf.io/d74vt/.

Our results revealed a developmental transition in ability to accurately detect pedagogical questions on our task. A one-sample *t*-test showed that whereas 4-year-olds performed at chance level on this task (*M* = .49, *t*(31) = 1.25, *p* = .22), 5-year-olds (*M* = .54, *t*(32) = 2.04, *p* = .05, *d* = .36), 6-year-olds (*M* = .56, *t*(32) = 3.44, *p* = .002, *d* = .6) and adults (*M* = .64, *t*(31) = 6.13, *p* < .001, *d* = 1.1) performed significantly above chance level. Thus, at least from age 5 or 6, children and adults can explicitly identify the pedagogical intent of a question on the basis of prosody alone. Not surprisingly, a regression analysis showed that Age was a significant predictor of accuracy on this task both with the Adults included (*p* < .001) and excluded (*p* = .007) from the analysis.

The higher accuracy rate of older children and adults is not surprising considering the difficulty of the task. Recall that participants were given no contextual cues and were relying on prosodic information only. Prosodic information, on the other hand, is inherently noisy, because it varies naturally across different individuals even when they are stating the same questions with the same intent. Therefore, to isolate and explicitly identify prosodic cues that differentiate pedagogical from information-seeking questions would, at the very least, require an adequate understanding of the task at hand, an understanding that the asker could be knowledgeable or ignorant about the answer to the question, as well as some form of metacognitive monitoring (i.e., reasoning about one’s own perceptual experiences, such as “this question sounded to me like it was asked by a knowledgeable teacher”).

A cross-experiment comparison between Experiments 1a and 2 shows that the effect size in Experiment 2 (*M* = .64, *d* = 1.1) is higher than that in Experiment 1a (*M* = .53, *d* = .62). One possibility that can explain this difference is that judging which question is pedagogical when it is juxtaposed to an information-seeking question (as presented in the procedure or Experiment 2) may be easier than hearing the question in isolation (as presented in the procedure of Experiment 1a). However, a more likely explanation for the higher performance in Experiment 2 is that the stimuli in Experiment 2 were better exemplars of pedagogical and information-seeking questions. As described in the Materials and Methods, the stimuli for Experiment 2 were chosen by selecting recordings that received high accuracy scores from the Mechanical Turk participants in Experiment 1a. Thus, it is perhaps not surprising that participants found it easier to differentiate pedagogical from information seeking questions in Experiment 2 even though they heard each question on a one-off basis, and the questions were spoken by multiple speakers who naturally differ across many prosodic dimensions.

Taken together, the results from Experiments 1 and 2 show that listeners can make an explicit inference about the pedagogical or information-seeking intent of questions by relying on prosodic cues only. These results show that there is a decoding machinery of prosodic cues already in place at least from age 5. Prosodic cues are taken as input, and inferences about the question’s intent and the speaker’s knowledge state are the output.

The results from Experiments 1 and 2 show that prosody is a viable cue to pedagogical intent. However, they do not support the conclusion that the prosodic cues marking pedagogical intent are applied in everyday spontaneous speech. There are at least two ways in which the results of Experiments 1 and 2 could have occurred in the absence of pedagogical acoustic cues in natural speech. The first is that participants could have identified the “canonical” sounding question and then concluded that the other “non-canonical” question must be a pedagogical question. On this view, the prosody of pedagogical questions could be just about anything, as long as it is somehow different from the canonical prosody of information-seeking questions. Another possibility is that there are some prosodic cues that “sound” pedagogical, while no one is actually using them in everyday life. For example, stomping with one leg while looking at a wristwatch in a theatrical setting signals anger that someone is late, and the audience can understand this. However, such behaviors are rarely exhibited in everyday settings (Swingley, personal communication, January 2021). Along the same lines, the pedagogical acoustic cues might be recognized as such, without ever being used in spontaneous communication. To address this limitation, in Experiment 3, we asked whether parents spontaneously vary their prosody when asking pedagogical versus information-seeking questions.

### Experiment 3: Parents Spontaneously Vary the Prosody of Pedagogical Questions

Experiment 3 consisted of two empirical investigations: a “generation” task which provided naturalistic, semi-spontaneous questions from parents in prompted pedagogical and information seeking contexts, and an evaluative task where adults evaluated the questions. Participants in the generative portion of Experiment 3 were parent-child pairs (*N* = 35 pairs; Child Mean Age = 95.39 months; range = 74 to 119 months). The children’s age was deliberately chosen to fall at the border or outside the range when parents use child-directed speech. The point of this was to show that the prosody of pedagogical questions is somewhat orthogonal from the prosody of child-directed speech. Pairs were recruited from across the United States and participated in synchronous sessions over Zoom.

The parents who participated in this study had no knowledge of the hypothesis being tested; there was no mention of anything relating to the prosody of questions. Rather, parents and children were invited to play two teaching games. In one of the games the child was the teacher, and in the other game the parent was the teacher. In the Child-as-Teacher game, children were invited to put on headphones so that the experimenter could tell them about five fun facts without the parent hearing (e.g., that dolphins sleep with one eye closed, one eye opened). Next, parents were invited to ask (supplied, information seeking) questions about each one of the five fun facts (where the questions were provided by the experimenter to the parent). After parents asked all the information seeking questions, children taught the answer to the parent’s questions. In the Parent-as-Teacher game, parents were invited to put on headphones so that the experimenter could tell them about five fun facts. Next, parents were invited to ask (supplied, pedagogical) questions, before teaching their child what the answer was. The order of the games (Parent-as-Teacher First or Child-as-Teacher First) was counterbalanced across participants. Although we use “pedagogical” and “information seeking” to distinguish conditions and question-types here, parents were not given information about different types of questions or our goals prior to or during the study. To foreshadow our results, we furthermore note that this design provides a very stringent test of our hypothesis, considering that both the information-seeking and the pedagogical questions were produced in a teaching context, thereby likely biasing parents to produce pedagogical questions in both games.

Participants in the evaluative portion of Experiment 3 were 128 English speakers recruited on Mechanical Turk. The parent-child interactions produced a total of 292 audio files. We randomly assigned the 292 questions to 4 different sets (i.e, each set having 73 questions), such that each question in a set was evaluated by 32 participants (4 sets; each set was evaluated by 32 participants = 128 participants). Thus, each participant received 73 questions and was asked to rate them as: a) A pedagogical question or b) An information-seeking question.

The pre-registration details are available at https://aspredicted.org/gwjm-73vx.pdf. We note here that we pre-registered to record 32 parent-child pairs. However, because several families signed up simultaneously toward the end of the study, the final sample was 35 parent-child pairs. The raw data are available at https://osf.io/d74vt/.

On average, participants were able to discriminate questions generated by parents in pedagogical contexts from those in the information seeking context on the basis of prosody alone. The average accuracy score for discriminating pedagogical from information-seeking questions was *M*_*PQ*/*ISQ*_ = .53, which is significantly different from chance performance (*t*(127) = 4.95, *p* < .001, *d* = .44. However, participants’ performance was not uniform across the different orders. Inspection of Table 1 shows that Mechanical Turk participants, on average, accurately recognized all pedagogical questions asked by parents, regardless of whether they were asked in the Parent-as-Teacher First or the Child-as-Teacher First order. A paired *t*-test confirmed that the accuracy at identifying pedagogical questions was comparable across the two orders (*M*_*PARENT_FIRST*_ = .54 and *M*_*CHILD_FIRST*_ = .54; *t*(127) = .02, *p* = .98).

**Table 1.** Accuracy for discriminating pedagogical questions from information seeking questions across different orders.

	Pedagogical Parent as Teacher First	Pedagogical Child as Teacher First	Information-Seeking Parent as Teacher First	Information-Seeking Child as Teacher First
Accuracy	0.54*	0.54*	.56***	0.47

*Note*: One-sample *t*-test comparisons against chance.

Key: *p < .05, **p < .01, ***p < .001.

A departure from the predicted results was observed for the information-seeking questions in the Child-as-Teacher First order. A paired *t*-test showed that the accuracy at identifying information-seeking questions was significantly higher in the parent as teacher first order (*M*_*PARENT_FIRST*_ = .56) than in the child as teacher first order (*M*_*CHILD_FIRST*_ = .47) (*t*(127) = 5.27, *p* < .001). That is, when parents were supposed to produce information-seeking questions in the initial stages of the experiment, they instead produced questions that sounded more like pedagogical questions, according to the Mechanical Turk participants’ judgments. A plausible post hoc interpretation of this result is that when parents are in a teaching context—as they were in this study—they are biased to produce pedagogical questions even when they play a role of a learner. It appears that parents could override this bias, if they had a chance to ask pedagogical questions first, and then ask information-seeking questions in the later stages of the experiment. In conclusion, despite the order effects, on average, parents do spontaneously vary the prosody of pedagogical and information-seeking questions when talking to their child, and naive listeners can decode the intent of the question without having any context whatsoever in which the question was asked.

#### Prosodic Cues That Differentiate Pedagogical From Information-Seeking Questions.

The primary goal of this series of studies was to investigate whether the prosody of speech is a viable and an actual cue that differentiates pedagogical from information-seeking questions. Experiments 1 and 2 suggested that prosody is a viable cue of pedagogical and information-seeking questions, and Experiment 3 suggested that prosody is an actual cue that parents spontaneously use in interactions with their child. In other words, Experiments 1 through 3 were not designed to investigate the specific acoustic signatures of pedagogical and information-seeking questions, nor were they designed to gauge which acoustic cues influence the listeners’ classification decisions. Nevertheless, we begin exploring these questions by looking at acoustic features that differed systematically between pedagogical and information-seeking questions across the experiments.

We investigated acoustic features that have been shown to be important in child-directed speech (Fernald & Kuhl, 1987; Fernald & Simon, 1984), as well as acoustic features implicated in research on inquisitive semantics that has suggested that non-canonical pitch contours (Bartels, 1999) of wh- questions are associated with episodes where the asker has partial knowledge of the answer (Hedberg & Sosa, 2011). Here, we present two such acoustic features, namely duration and pitch contour at the end of the question. To do this analysis, all audio files were manually segmented and annotated at a word level. To make the manual annotation manageable, we chose a subsample from the 3200 audio files recorded in English for Experiment 1 by selecting the ones that were accurately rated by 3 or 4 (out of 4) Mechanical Turk participants on the pedagogical/information-seeking dimension. This yielded 143 pairs of perfectly matched adult-directed pedagogical and information-seeking questions and 180 perfectly matched child-directed pedagogical and information-seeking questions. Considering that we selected the highly rated—by MTurk participants—audio files, they can be considered to be prototypical examples of pedagogical and information-seeking questions (i.e., as defined by the naive listeners’ ratings). Finally, to explore whether parents spontaneously vary their prosody when asking pedagogical questions as speakers who produce simulated pedagogical questions, we did the same manual segmentation and annotation at a word level of all 292 audio files from Experiment 3.

#### Duration.

To investigate whether there is a difference in the duration between pedagogical and information-seeking questions, we first computed the duration by dividing the total duration in seconds of the utterance with the number of words in the utterance. Next, we standardized the duration within-speaker across pedagogical and information-seeking questions. Thus, a positive standardized score represents a longer duration, while a negative score represents a shorter duration.

**Figure 3.**
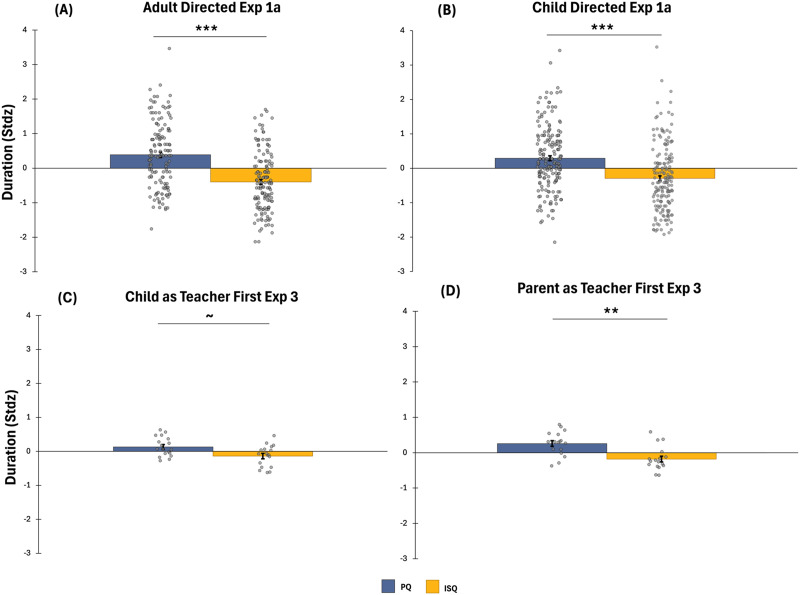
The average duration of pedagogical questions (PQ) is longer than the average duration of information-seeking questions (ISQ). Panels A and B present duration of pedagogical and information-seeking questions in Experiment 1a within adult directed and child-directed speech respectively. Panels C and D present duration of pedagogical and information-seeking questions in Experiment 3 across the two orders respectively. Error bars are standard errors.

We found that, on average, pedagogical questions are with a longer duration than information-seeking questions across all experiments. Figure 3 represents the standardized (within speaker) duration of pedagogical and information-seeking questions within Experiment 1a (adult-directed (Panel A) and child-directed (Panel B)) and within the two orders of Experiment 3 (Panels C and D respectively). As the graph suggests, pedagogical questions had a longer duration compared to information-seeking questions in Experiment 1a, both within adult-directed (*t*(141) = 11.18, *p* < .001, *d* = .94) and within child-directed speech (*t*(179) = 8.49, *p* < .001, *d* = .63). In Experiment 3, a repeated measures ANOVA with duration as a dependent variable, examined the effects of order (Child as Teacher First vs. Parent as Teacher First) and type of question (Pedagogical vs. Information-Seeking). The analysis revealed a main effect of type of question *F*(1, 33) = 11.23, *p* = .002, a main effect of order *F*(1, 33) = 4.33, *p* < .05, and a non-significant interaction (*p* = .45), suggesting that the duration was longer for pedagogical questions. The order effect is most likely driven by the longer duration of pedagogical questions in the parent as teacher first order, which is consistent with the finding reported above that the questions produced in the parent as teacher first order were easier to differentiate.

Although the pitch contour and other prosodic cues of Macedonian questions is likely different from that of the English language, the duration is language neutral. Since the stimuli were the exact same questions that could be compared directly, we computed the absolute duration of all questions recorded in Macedonian language. We found that the average duration of adult-directed pedagogical questions was *M* = 2.9 seconds, and it was 1.9 seconds for information-seeking questions. This difference was statistically significant (*t*(199) = 22.85, *p* < .001, *d* = 1.62). Similarly, within child-directed speech, the average duration of pedagogical questions recorded in Macedonian language was *M* = 3.04 seconds and it was *M* = 2 seconds for information-seeking questions, which was also a statistically significant difference (*t*(199) = 24.7, *p* < .001, *d* = 1.75). In summary, pedagogical questions are slower than information-seeking questions both when produced in laboratory settings as well as when spontaneously produced by parents during parent child interactions.

#### Pitch Contour.

To investigate the pitch contour at the end of the question, we computed a difference score of the average fundamental frequency (F0) of the last word and the average fundamental frequency of the next to last word. Since questions that call for a yes/no answer have a different contour than wh- questions, the only 7 questions that called for a yes/no answer were excluded from this analysis. If the pitch is dropping, as it does canonically in wh- questions, then the difference score would be a negative number. If the pitch is rising, then the difference score would be a positive number. Here, we present both the absolute difference scores across the experiments, as well as the difference scores that were standardized within-speaker and across pedagogical and information-seeking questions.

We found that, on average, the difference score of information-seeking questions was lower than the difference score of pedagogical questions. First, we present the results descriptively. The average difference score within child-directed speech in Experiment 1 was 9.59 HZ for pedagogical questions and it was −24.9 HZ for information-seeking questions. Within adult-directed speech, it was 14.25 HZ for pedagogical questions, and it was −32.54 HZ for information-seeking questions. In Experiment 3, however, the average difference score was 44.38 HZ for pedagogical and 49.34 HZ for information-seeking questions in the Child as Teacher First order. Notably, the absolute difference score was on average higher in information-seeking than in pedagogical questions in the Child as Teacher First order. This result is consistent with the finding that Mechanical Turk participants had difficulty differentiating pedagogical from information-seeking questions in this order. Finally, the average difference score was 52.3 HZ for pedagogical and 16.73 HZ for information-seeking questions in the Parent as Teacher First order.

**Figure 4.**
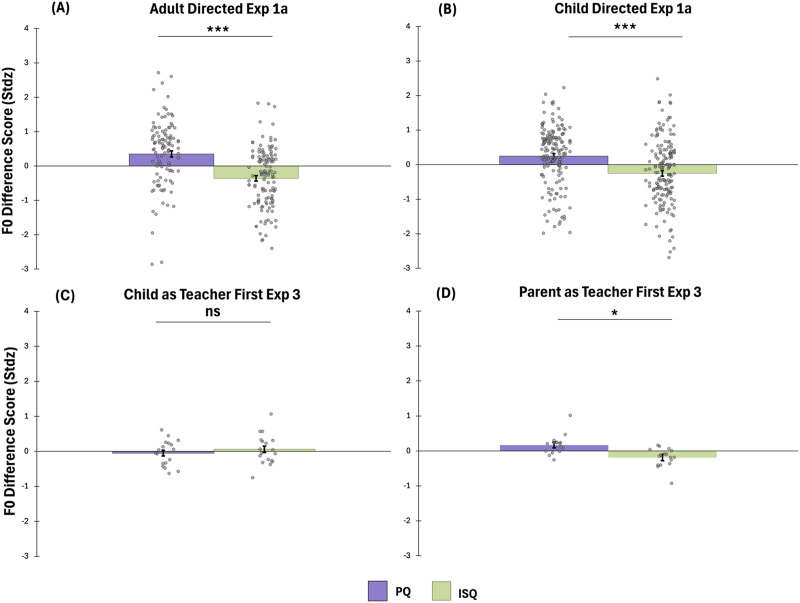
Pedagogical questions (PQ) on average have a rising pitch at the end of the sentence compared to information-seeking questions (ISQ) that have a canonical falling pitch. Panels A and B present F0 difference scores of the last and the before last word of pedagogical and information-seeking questions in Experiment 1a within adult directed and child-directed speech respectively. Panels C and D present F0 difference scores of pedagogical and information-seeking questions in Experiment 3 across the two orders respectively. Error bars are standard errors.

We formally tested these differences by comparing the standardized scores. Figure 4 represents the standardized (within speaker) average fundamental frequency difference score for pedagogical and information-seeking questions in Experiment 1 (adult-directed (Panel A) and child-directed (Panel B)) and within the two orders of Experiment 3 (Panels C and D respectively). As the graph suggests, in Experiment 1, pedagogical questions had an increasing (noncanonical) pitch contour at the end of the question both within adult-directed (*t*(118) = 6.3, *p* < .001, *d* = .58) and within child-directed speech (*t*(162) = 4.85, *p* < .001, *d* = .38). In Experiment 3, a repeated measures ANOVA with F0 difference score as a dependent variable, examined the effects of order (Child First vs. Parent First) and type of question (Pedagogical vs. Information-Seeking). The analysis revealed non-significant main effects and a significant Order × Type of Question interaction (*p* = .04). A follow up simple effects analysis revealed that the difference score for pedagogical questions was higher than that of information-seeking questions in the Parent as Teacher First order only (*p* = .03), suggesting that the average standardized difference score of pedagogical questions was higher than the difference score of information-seeking questions, which is in turn consistent with the pitch of most pedagogical questions rising at the end of the sentence, while the pitch of information-seeking questions is falling at the end of the sentence. The difference between the two types of questions was not significant in the Child First order (*p* = .5). Note that this is consistent with the ratings of the questions by the Mechanical Turk participants who could accurately distinguish pedagogical from information-seeking questions recorded in the Parent as Teacher First order but had difficulty with this task in the Child as Teacher First order.

## GENERAL DISCUSSION

While questions have been an instrument for discovery, learning, and teaching, recent research has shown that pedagogical questions specifically, as opposed to information seeking questions, enable powerful inferences (Yu et al., 2019). However, how different types of questions are marked and interpreted in day-to-day interactions is not well understood. In the present series of studies, we tested if the prosody of questions is a viable and actual mechanism that may disambiguate pedagogical from information-seeking intent. We found that prosody is a viable mechanism: naive adult listeners accurately differentiate between pedagogical and information-seeking questions by relying solely on the prosody of speech, even when the questions are spoken in a foreign language; and young children at least from age 5 years have a capacity to explicitly identify which asker is knowledgeable and asks the question with an intention to teach on the basis of prototypical prosody alone. In addition, we found that the prosody of spontaneous speech produced by parents in a teaching versus an information-seeking context are recognized as pedagogical or information-seeking questions respectively by naive listeners. In summary, both adults and children can recognize deliberately produced pedagogical and information-seeking questions on the basis of prosody alone, and parents spontaneously modulate their prosody when interacting with their child in pedagogical and information-seeking contexts.

The prosodic cues that mark pedagogical and information-seeking questions are different from the prosodic cues that mark child-directed and adult-directed speech. This is supported by the results from Studies 1 and 2 showing that naïve listeners can differentiate between pedagogical and information-seeking questions both when they are spoken with child-directed or adult-directed prosody. Furthermore, in Study 3, naïve listeners could differentiate between pedagogical and information-seeking questions even though all questions were directed to children only. However, although the pedagogical/information-seeking distinction is independent from the child-directed/adult-directed distinction, there seems to be some overlap between the two. For example, the longer duration of pedagogical questions, which may be helpful for emphasizing the content of the utterance in a pedagogical context, is also a feature shared with child-directed speech (e.g., Fernald & Simon, 1984; Kempe et al., 2010). This may explain why the naïve listeners in Study 1 were less likely to accurately rate child-directed information-seeking questions as information-seeking questions. An alternative but not mutually exclusive explanation of the same result, however, is that whenever naïve listeners recognize a child-directed speech on the basis of various prosodic cues, they are biased to think that the question is pedagogical because they believe that children are more likely to receive pedagogical questions than adults. Indeed, the Signal Detection analysis showed that listeners apply a more liberal criterion when detecting pedagogical questions within child-directed speech than within-adult-directed speech, which leads to higher Hits of pedagogical questions, but also a higher rate of False Alarms (i.e., saying that they heard a pedagogical question in cases when they heard an information-seeking question). In summary, although pedagogical questions may share some prosodic features with child-directed speech, and although pedagogical questions may be more frequently used when talking to children than to adults, the distinction between pedagogical and information-seeking questions is independent from the distinction between child-directed and adult-directed speech.

Relatedly, the function of pedagogical prosody seems to be different from the function of child-directed prosody. Specifically, child-directed prosody seems to be more general in terms of the contexts in which it is used, and more specific in terms of who it is directed to. Although child-directed prosody—also smiling, eye-contact, or contingent behavior—may be interpreted by young children as cues to a beginning of a pedagogical episode (Csibra & Gergely, 2009; Gergely et al., 2007), child-directed prosody is also associated with language learning (e.g., Eaves et al., 2016; Nencheva et al., 2021; Rowe, 2008; Thiessen et al., 2005; Trainor & Desjardins, 2002; Vallabha et al., 2007), attentional/affective functions (Vallabha et al., 2007), and it serves the function of emotion expression and emotion regulation (Trainor et al., 2000; see Saint-Georges et al., 2013 for review). Conversely, pedagogical prosody seems to be deployed in pedagogical contexts only, and it could be directed both at children and adults, suggesting that the primary function of pedagogical prosody is to signal the pedagogical intent of the speaker to recipients of all ages. In summary, pedagogical prosody seems to be more specific in terms of the contexts in which it is used, and more general in terms of who it is directed to.

Would a specific prosodic marker for pedagogical questions be useful in early childhood when one might assume that most questions that children receive are pedagogical? The answer to this question is yes. Contrary to the assumption that most questions are pedagogical, and although pedagogical questions have been increasing over the last several decades, according to some estimates of UK and US populations, pedagogical questions make up just 42% of questions (though they are more common in families from higher SES; Yu et al., 2019) . Moreover, the number of pedagogical questions decreases with age (Yu et al., 2019). Given that there is a great variability in terms of types of questions that children receive in early childhood and the inherent ambiguity of contextual cues suggests that a specific prosodic marker of pedagogical intent could be very useful for discerning the intent of the received question in early childhood.

What is the mechanism by which prosody signals pedagogical intent? One possibility—indirectly supported by the present studies—is that prosody allows for an inference about the epistemic state of the asker, which in turn allows for an inference about the pedagogical or the information-seeking intent of the question. That is, a canonical falling intonation at the end of wh- questions in English language signals inquisitive intent. A non-canonical rising intonation at the end of wh- questions, however, signals that the asker has either a partial or complete knowledge of the answer to the question (Hedberg & Sosa, 2011). If the speaker knows the answer to the question, but is still asking it, then the listener has a basis to draw an inference that the intent of the question is pedagogical rather than inquisitive (Yu et al., 2018). This interpretation of the results of the present studies could be further tested in future studies in at least two different ways. The first possibility is to investigate Yes—No questions that have a reversed intonation compared to wh- questions. The prediction would be that a noncanonical falling intonation at the end of Yes—No questions would be interpreted as pedagogical by naïve listeners, and that speakers asking pedagogical Yes—No questions in a pedagogical context would use non canonical falling intonation. The second possibility is to investigate the effects of canonical and non-canonical prosody of declarative statements on learning. In that case, a noncanonical rising intonation at the end of a declarative statement would signal uncertainty in one’s knowledge (Gunlogson, 2004), which may undermine its pedagogical thrust. Whether such non canonical intonation influences the recipients’ inferences and learning outcomes is an open question.

The present study showed that young children can use the prosody of speech to make an explicit inference about the pedagogical or non-pedagogical intent of the question. As noted in the Introduction, prior research has shown that, when asked a pedagogical question as opposed to an information-seeking question, young children behave differently (Bonawitz et al., 2020) and show superior learning outcomes (Jean et al., 2019; Yu et al., 2018). Taken together, these findings suggest that young children would likely behave differently and show different learning outcomes if exposed to pedagogical or non-pedagogical prosody. For example, a child who infers that the question is information-seeking may be quicker to answer “I don’t know” than a child who infers that the question is pedagogical. That is, a child who infers pedagogical intent may spend more time and effort in a search for an answer. They may do so because pedagogical prompts suggest there is information to be learned, that the information is learnable, and that the asker believes the child is capable of that discovery. Furthermore, information that is discovered as a result of pedagogical prompts may also be considered “more important” and thus more likely to be encoded. Indeed, it has been suggested that expectation of information may trigger brain responses that support learning and memory (e.g., see Begus & Bonawitz, 2020). These predictions could be tested directly in future research. Moreover, future research could investigate the interplay between cues to pedagogy and children’s ability to infer pedagogical intent (e.g., as in Bass et al., 2024). Although there are proposals that the ability to infer pedagogical intent is present early in infancy and it is probably not learned (Csibra & Gergely, 2011), it remains unclear how the social and linguistic inputs influence and finetune children’s ability to infer pedagogical intent.

A related issue is the developmental trajectory of children’s sensitivity to prosodic cues that differentiate between pedagogical and information-seeking questions. The present study suggests that at least from age 5, listeners can explicitly identify which questions have a pedagogical or information-seeking intent. These findings somewhat mirror the findings that children can explicitly detect the state of *disbelief* in a speaker on the basis of facial and prosodic cues between the ages of 3 and 5 with a clear developmental improvement with age (Armstrong et al., 2018). Similarly, children’s own production of prosodic cues that signal uncertainty in one’s beliefs emerges also between the ages of 3 and 5 and continues to develop in later childhood (Hübscher et al., 2019). However, it should be emphasized that all these tasks require an explicit detection of prosodic cues. In order to make such judgments, however, one needs to be metacognitively aware of the task at hand, needs to decide how a particular question sounds, and needs to verbally report the judgment. It is likely that these metacognitive factors contributed to the difficulty of the tasks and are the reason why 4-year-old children were performing at chance level in the present study. Developing new sensitive implicit measures could address this concern. One possibility would be to investigate the neural signatures of listeners who are receiving either pedagogical or information-seeking questions. For example, it has been suggested that EEG theta oscillations are associated with information expectation, learning, and memory formation (Begus & Bonawitz, 2020, 2024), thereby making this neural signature a potential neural marker of engaging in pedagogical episodes. The development of such new measures could shed further light on the developmental trajectory of children’s sensitivity to cues that signal pedagogical episodes.

One limitation of the present series of studies is the relatively modest effect sizes across all experiments. In addition to the metacognitive demands that made the tasks difficult, it should be noted that the stimuli used in the present studies have been stripped of all other contextual cues. Of course, this was done intentionally to allow us to control for the confounds of other cues to intent. Nonetheless, both producing and hearing prosody stripped from all other information is ecologically invalid. In day-to-day experiences, speakers produce questions in the context of an interaction and listeners likely rely on multiple sources at once, where converging information from multiple sources may reduce the noise and increase the signal from each individual source of information (e.g., acoustic, visual, or contextual). Despite this limitation, however, the present studies are the first evidence that pedagogical questions are acoustically marked by askers and appropriately interpreted by naïve listeners.

In conclusion, the intent of questions enables powerful inferences about the world. The present studies showed that one relatively unexplored factor, namely the prosody of speech, supports inferences about a question’s intent. Both young children and adults can infer a pedagogical or an information-seeking intent on the basis of prosody alone. Moreover, parents spontaneously vary the prosody of speech when in pedagogical and information-seeking contexts, and naïve listeners can accurately categorize the speech produced in those contexts. These findings raise many new questions about the potency of pedagogical questions, about the interaction of prosodic and other cues, about the variability of pedagogical prosodic cues across different languages, cultures, and different communities, about the effects of pedagogical prosodic cues on learning and educational outcomes, or about the importance of naturalistic prosodic signals implemented in educational technologies. No doubt, as these questions are answered, new ones will arise. As Socrates would argue, through questioning we discover our ignorance, thus moving closer to true wisdom.

## MATERIALS AND METHODS

### Experiment 1a

Participants were 128 adults who were recruited from the US on Amazon’s Mechanical Turk platform. The research was approved by the Rutgers University, Newark’s Institutional Review Board. The experiment was performed in accordance with all ethical guidelines and regulations. Participants signed an informed consent form before participating. The participants’ average age was 39 years (range = 19–72; *SD* = 12.73). Two participants were excluded and replaced because they completed the experiment in a timeframe of 338 and 270 seconds respectively, which is incommensurable with listening to the audio clips. Sixty-five participants identified as men, sixty-two as women, and one participant identified as non-binary genderqueer. Sixty-five participants identified as parents and forty said that they had worked with children in some professional capacity (e.g., a teacher, a nanny, or similar).

The stimuli were recorded by 8 (four women and four men) native speakers of American English. The speakers come from diverse ethnic and cultural backgrounds, and have extensive experience interacting with children (i.e., as parents or developmentalists). Specifically, of the 8 speakers, 5 are White, not of Hispanic origin, 1 is Asian/Pacific Islander and White, 1 is Black/African American and Hispanic, and 1 is Black/African American. Of the 8 speakers, 3 are parents/guardians, and all 8 speakers are developmental psychologists with extensive experiences working with children. The stimuli consisted of 100 short questions covering the physical, biological, and social domains (e.g., “Is the speed of light the same in all mediums?”; “How do dolphins sleep?”; “Why do people gossip?”). The majority of the questions (i.e., 93 out of 100) were wh- type questions, and only 7 questions called for a yes or no answer. The list of all questions and the context that was given to differentiate between PQs and ISQs can be found at https://osf.io/d74vt/.

Each speaker recorded all 100 questions in four different ways: 1) as an adult-directed pedagogical question; 2) as adult-directed information-seeking question; 3) as a child-directed pedagogical question; and 4) as a child-directed information-seeking question. Before the recording, the speakers were told descriptively what a pedagogical question is (i.e., a question where the asker knows the answer and asks the question with an intention to teach), what an information-seeking question is (i.e., a question where the asker does not know the answer and asks the question with an intention to learn), and what child-directed and adult-directed speech is. An important aspect of this design is that it allowed a comparison of the exact same questions that were recorded as pedagogical and as information-seeking questions. Importantly, the speakers were not told how the stimuli should sound. Each speaker was left on their own to decide how child-directed and adult-directed PQs and ISQs should sound. To aid the recording of the stimuli, each question was preceded by a context sentence providing a pedagogical or an information-seeking context. For example, the information-seeking context sentence that preceded the question “How do dolphins sleep” was “I have no idea how animals that live in the sea sleep. Do you know …” The pedagogical context sentence for the same question was: “I learned about this at the aquarium a few days ago. Do you know …” Importantly, the ending of the provided context (e.g., “Do you know …”) was the same across pedagogical questions and information seeking questions. The speakers read the questions in blocks. The order of blocks was counterbalanced across speakers and the order of questions within each block was randomized.

We created 4 different surveys from the 400 questions recorded by each speaker. Each of the 4 surveys included 100 questions, 25 of each question type. Each survey was presented to 4 participants on Mechanical Turk (4 participants per survey × 4 surveys per speaker × 8 speakers = 128 Mechanical Turk participants).

The surveys were prepared and administered via Qualtrics. After answering a few demographic questions, participants read a description of what pedagogical questions are and what information seeking questions are, and also a description of what is meant by child-directed and adult-directed questions. Following that short training, participants were tasked with listening to the audio clips (a total of 100) and answering two questions about each audio clip: 1) Is the question you heard: a) A Pedagogical Question; b) An Information Seeking Question; and 2) Is the question you heard: a) A Question Directed to a Child; b) A Question Directed to an Adult. The 100 audio clips were randomized within each survey. The order in which the two questions appeared (PQ vs. ISQ or CD vs. AD) and the order in which the two alternative answers appeared after each audio clip were counterbalanced.

### Experiment 1b

Participants were 128 adults who were recruited from the US on Amazon’s Mechanical Turk platform. The research was approved by the Rutgers University, Newark’s Institutional Review Board. The experiment was performed in accordance with all ethical guidelines and regulations. Participants signed an informed consent form before participating. Six participants were excluded and replaced because they reported that they could understand Macedonian language. The participants’ average age was 39 years (range = 24–74; *SD* = 10.61). Sixty-eight participants identified as men, fifty-nine as women, and one participant identified as non-binary genderqueer. Sixty-seven participants identified as parents and forty-six said that they had worked with children in some professional capacity (e.g., a teacher, a nanny, or similar).

The stimuli were the same as in Study 1, except they were translated and recorded in Macedonian language by 2 speakers (1 man and 1 woman). The stimuli were recorded by native speakers of Macedonian language. The two speakers, however, live and work in the US. Just like the English language stimuli, these stimuli were recorded by providing context sentences and imagining that the question is pedagogical or information-seeking and that the question is directed to an adult or a child.

As in Experiment 1a, four different surveys were created from the 400 questions recorded by the speakers. Each survey contained 100 questions, 25 of each question type. Each survey was presented to 16 participants on Mechanical Turk (16 participants per survey × 4 surveys per speaker × 2 speakers = 128 Mechanical Turk participants).

The procedure and the questions in the survey were equivalent to those in Experiment 1a.

### Experiment 2

Participants were 130 four-, five-, and six-year-old children, as well as adults. Broken down by age group, the participants were 32 four-year-olds (*M* = 53.97 months; *SD* = 3.34), 33 five-year-olds (*M* = 65.85 months; *SD* = 4.06); 33 six-year-olds (*M* = 79.06 months; *SD* = 3.87) and 32 undergraduate students at Rutgers University, Newark (25 women).

The research was approved by the Rutgers University, Newark’s Institutional Review Board. The experiment was performed in accordance with all ethical guidelines and regulations. Parents of children who participated in the study read and signed an informed parental consent form. Children also assented to participating in the research study. Adult participants signed an informed consent form before participating.

A total of 77 families whose children participated in this study completed the optional demographic survey. Children were recruited from across the United States and participated via Zoom. The breakdown of the reported race/ethnicity is as follows: 67.5% identified as White Non-Hispanic, 10.4% identified as Asian/Pacific Islander, 7.8% identified as multiracial/multiple ethnicities, 3.9% identified as Black/African American, 3.9% identified as Hispanic, 2.6% identified as South Asian/Indian, and 2.6% identified as American Indian/Alaska Native. Children’s parents in this sample were on average highly educated with 91% of all parents having AB/BA or advanced degrees. The breakdown of the reported household income is as follows: 9.1% reported income less than $40,000, 10.4% reported income between $40,000 and $60,000, 15.6% reported income between $60,000 and $100,000, and 59.7% reported income more than $100,000.

All but one of the undergraduate students who participated in this study completed the optional demographic survey. The breakdown of the reported race/ethnicity is as follows: 27.3% identified as Hispanix/Latinx, 21.2% identified as Black/African American, 18.2% identified as Asian/Pacific Islander, 12.1% identified as White, not of Hispanic origin, 9.1.% identified as South Asian/Indian, and 9.1% identified as Other.

The stimuli for this study were constructed from a subsample of Experiment 1a’s audio recordings. Recordings that received high accuracy scores from the Mechanical Turk participants in Experiment 1a were chosen for this study. Recall that the audio recordings in Experiment 1a were created by 8 speakers (4 men and 4 women). Therefore, there were 6 possible contrasts within gender (speaker 1 vs. 2, 1 vs. 3, 1 vs. 4, 2 vs. 3, 2 vs. 4, and 3 vs. 4). We used all possible contrasts twice for a total of 12 contrasts within gender, which generated a total of 24 contrasts. The 24 contrasts consisted of 24 unique questions. Whether any given speaker spoke with pedagogical, or information-seeking prosody was counterbalanced both within and across participants. For example, for the speaker 1 vs. 2 contrast, speaker 1 spoke with pedagogical prosody on the first question and with information-seeking prosody on the second question within-child for one half of the participants. For the other half of the participants, speaker 1 spoke with information-seeking prosody on the first question, and with pedagogical prosody on the second question. In addition, whether children first heard a pedagogical or an information-seeking question on any given trial and whether the voice with pedagogical prosody was on the left or on the right side of the screen was also counterbalanced. The order in which the contrasts were presented to children and adults was randomized, with the constraint that the contrasts of male and female voices alternated. All questions in the 24 contrasts were spoken with a child-directed prosody.

All data collection was conducted synchronously over Zoom. The procedure was the same for both children and adults. At the beginning of the procedure, participants were told that they will need to make guesses by listening carefully. First, participants received two warm up trials, designed to familiarize them with the structure of each trial. While sharing the screen, the experimenter showed drawings of 4 individuals and said: “Here I have some friends that I made drawings of. Some of my friends speak loudly, and some of them speak quietly. Some of them speak fast and some of them speak slow. Now two of these people will ask you the same question, but only one of the two people will speak slowly and the other one will speak fast. Your job is to guess who speaks fast. Let’s first hear these two. First, we’ll hear the one in the brown shirt, the one who has a square around her/him. Okay. Next, we’ll hear the one in the pink shirt, the one who has a square around her/him. So, can you tell me? Who spoke fast? The one in the [some color short with or without stripes] or the one in the [some color shirt with or without stripes]?” After the familiarization trials, the experiment showed a screen with drawings of 8 individuals and said: “Here I have some other friends. Some of these friends know a lot about some things because they teach about those things, but they don’t know much about other things because they are still learning about those things. For example, some of my friends know a lot about animals because they teach about animals, but those who know a lot about animals don’t know much about other things, for example … They don’t know much about how airplanes fly because they are still learning about that. And those who know a lot about how airplanes fly, don’t know much about animals. Now two people will ask you the same question, but only one of the two people is teaching and knows the answer to the question they asked, and the other person is learning and does not know the answer to the question they asked. Your job is to listen carefully to how these people sound and guess who is teaching and knows the answer to the question they asked.” Next, the structure of each trial was the same as the warmup trials. The pairs of speakers were presented on the screen on each of the 24 test trials. The “speaker” who spoke had a rectangle frame around her/him while speaking. After participants have heard both speakers, they were asked: “Who do you think is the teacher? Who knows the answer? The person in [some color shirt with or without stripes] or the person in [a different color shirt with or without stripes]?

### Experiment 3

Participants were 128 adults who were recruited from the US on Amazon Mechanical Turk platforms. The research was approved by the Rutgers University, Newark’s Institutional Review Board. The experiment was performed in accordance with all ethical guidelines and regulations. Participants signed an informed consent form before participating. The participants’ average age was 39.89 years (range = 20–74; *SD* = 13.01). Seventy-five participants identified as men, fifty-two as women, and one participant identified as non-binary. Fifty-three participants identified as parents and twenty-eight said that they had worked with children in some professional capacity (e.g., a teacher, a nanny, or similar).

To construct the stimuli, we conducted a separate study with 35 parent-child pairs who participated in synchronous sessions on Zoom. The experimental procedure was approved by the Rutgers University, Newark’s Institutional Review Board. Parents of children who participated in the study read and signed an informed parental consent form. Children also assented to participating in the research study. Due to experimenter error, we did not record the child’s age of 2 participants. The average age of the remaining 33 children who participated in the study was *M* = 95.39 months; *SD* = 14.27 months; range = 74 to 119 months. Five parent-child pairs were excluded and replaced. Three pairs were excluded, as defined in the pre-registration, because the parent’s first language was not English, and two pairs were excluded due to technical difficulties (i.e., ambient noise and malfunctioning headphones).

A total of 33 families who participated in this study completed the optional demographic survey. Parent-child pairs were recruited from across the United States and participated via Zoom. The breakdown of the reported race/ethnicity is as follows: 57.6% identified as White Non-Hispanic, 15.2% identified as Asian/Pacific Islander, 6% identified as multiracial/multiple ethnicities, 9.1% identified as Black/African American, 3% identified as Hispanic, 3% identified as American Indian/Alaska Native, and 6.1% identified as Other. A total of 69.7% parents in this sample had an AB/BA or advanced degrees. The breakdown of the reported household income is as follows: 12.1% reported income less than $40,000, 18.2% reported income between $40,000 and $60,000, 24.2% reported income between $60,000 and $100,000, and 39.4% reported income more than $100,000.

The parent-child pairs were invited to play two different games: i) a parent-teacher game where the parent was going to be the teacher and ii) a child-teacher game where the child was going to be the teacher. Neither the parent, nor the child, knew that the goal of the experiment was to investigate the prosody of questions. This goal was revealed only at the very end of the experiment. At the beginning, the experiment was introduced as a teaching and learning game. The order in which the two games were presented was counterbalanced across participants. In the parent-teacher game, the parent was invited to put on headphones, so that only the parent could hear about some fun and obscure facts. In the child-teacher game, the child was invited to put on headphones, so that only the child could hear about some fun and obscure facts. There were 10 fun facts (i.e., five fun facts presented within each game). The fun facts were organized in two blocks of 5. The order in which the blocks of fun facts were presented was counterbalanced across participants and across games. An example of a fun fact is where the ears of a cricket are? The answer: on their legs. The full details of the design and the list of fun facts can be found at https://osf.io/d74vt/.

In the parent-teacher game, after the experimenter explained the games and after the experimenter told the parent about the five fun facts, the experimenter said: “So now, on the screen I’ll show questions that your parent can read and ask you like teachers ask questions, and then after they ask you, you can answer, and then your parent will teach you what the answer is. So, [name of parent], you should now know the answers to these questions. Please read each question to yourself first (silently), and then turn to your child and ask the question out loud so that you can teach your child the information that follows.” Provided that for these questions, the parent knew what the answer was, and the question was asked with the intent to teach something, we considered these questions to be *pedagogical questions*.

In the child-teacher game, after the experimenter explained the games and after the experimenter told the child about the five fun facts, the experimenter said: “So now, on the screen I’ll show questions that your parent can read and ask you, and after they ask you, you can teach your parent what the correct answer is. Okay? So, [name of parent], you will probably not know the answers to these questions. Please read each question to yourself first silently, and then turn to your child and ask the question out loud so that your child can teach you.” Provided that for these questions, the parent did not know what the answer was, and the question was asked with the intent to learn something, we considered these questions to be *information-seeking questions*.

As described in the pre-registration, we asked parents at the end of the study if they knew the answers to the fun facts before the study began. The fun facts to which parents knew the answers to, and were supposed to be information-seeking questions, were excluded from the analysis. In addition, recordings where there was ambient noise or technical interruptions were also excluded. Of the 350 questions recorded in the study, during pre-processing, 58 audio recordings were excluded on the basis of those criteria. Thus, 292 audio recordings were generated in the parent-child interaction study.

Because 292 audio recordings is a large number for any participant on Mechanical Turk to rate, we randomly split the 292 recordings to 4 sets of 73 audio recordings. Each of these sets was then presented via Qualtrics to 32 participants on Mechanical Turk, for a total of 128 participants (4 sets × 32 participants = 128). After answering a few demographic questions, participants read a description of what pedagogical questions are and what information seeking questions are (same as in Experiment 1). Following that short training, participants were tasked with listening to the audio clips (a total of 73) and answering the following question about each audio clip: Is the question you heard: a) A Pedagogical Question; b) An Information Seeking Question. The 73 audio clips were randomized within each survey. The order in which the two alternative answers appeared after each audio clip were counterbalanced.

## ACKNOWLEDGMENTS

We are grateful to Michael LaSorsa, Liang Yan, and Nicholas Mcnab for their help with data collection and coding. We are thankful to the families and the Mechanical Turk participants without whom this research would have been impossible.

## FUNDING INFORMATION

This research was supported by the National Science Foundation, Education and Human Resources to EB & PS (1660885), National Science Foundation EAGER: Science of Learning & HNDS-R to EB & PS (2121842), and Jacobs Foundation CRISP award to EB. (No fund number associated for Jacobs.)

## AUTHOR CONTRIBUTIONS

IB: Writing – original draft; Writing – review & editing. PS: Writing – review & editing. EB: Writing – review & editing.

## DATA AVAILABILITY STATEMENT

The raw data of all three experiments presented in this paper are available at https://osf.io/d74vt/.
